# When the Part Mirrors the Whole: Interactions Beyond “Simple Location”

**DOI:** 10.3389/fpsyg.2020.523885

**Published:** 2021-01-22

**Authors:** Alex Gomez-Marin, Juan Arnau

**Affiliations:** ^1^Behavior of Organisms Laboratory, Instituto de Neurociencias CSIC-UMH, Alicante, Spain; ^2^Facultad de Filosofía y Letras, Universidad de Granada, Granada, Spain

**Keywords:** simple location, internal relations, misplaced concreteness, process philosophy, Alfred North Whitehead

## Abstract

Reductionism relies on expectations that it is possible to make sense of the whole by studying its parts, whereas emergentism considers that program to be unattainable, partly due to the existence of emergent properties. The emergentist holistic stance is particularly relevant in biology and cognitive neuroscience, where interactions amongst system components and environment are key. Here we consider Alfred North Whitehead's philosophy as providing important insights to metaphysics of science in general, and to the reductionism vs. emergentism debate in particular. An appraisal of Whitehead's perspective reveals a difficulty shared by both approaches, referred to him as “simple location”: the commitment to the idea that the nature of things is exhausted by their intrinsic or internal properties, and does not take into account relations or dynamic interactions denoting “togetherness.” In a word, that things are simply where they are. Whitehead criticizes this externalist ontological perspective in which each interacting element exists, and can be thought, without essential reference to other elements. The aim of this work is to uncover such a stance, particularly in the context of dynamical systems, and to show its shortcomings. We propose an alternative relational approach based on Whitehead's notion of “internal relations,” which we explicate and illustrate with several examples. Our work aims to criticize the notion of simple location, even in the framework of emergentist accounts, so as to contribute to a “relational turn” that will conceive “inter-identities” as “intra-identities” in which interactants are not enduring substances, but internally related processes. In sum, we argue that the notion of internal relations has a strong theoretical power to overcome some fundamental difficulties in the study of life and mind.

“*Berkeley afirma: Sólo existen las cosas en cuanto se fija en ellas la mente. L*í*cito es responderle: S*í*, pero sólo existe la mente como perceptiva y meditadora de cosas.”*

(Borges, 1925)

“*It has been usual, indeed, universal, to hold that spatio-temporal relationships are external. This doctrine is what is here denied.”*

(Whitehead, 1925).

## Introduction

It seems common sense to affirm that the world is made of discrete, independently existing objects. When we look around we see objects all over the place: pens, chairs, and trees. This everyday experience, when formally articulated as a philosophical system, corresponds to “substance metaphysics.” Namely, the presupposition that reality is like a building and that, as such, it is made of building blocks. The quest of the physicist and the philosopher is then to find out about those tiny building blocks, inquiring about the smallest of objects, in order to identify and characterize the constituents of reality.

However, at the very microscopic level, such bits of matter have not been found. Quite the contrary, the quantum physicist has bumped into an exotic garden of incredible particles which, when inspected even more closely, dissolve into energy fields. Once determined to come across the ultimate pellets of the real (the old “atom” idea of the Greeks), twentieth-century scientists realized that it is more appropriate to think of them as expressions of activity.

In philosophy, such a change of paradigm exists and it has a name: it is called “process metaphysics.” Having a long history (more details further below), and epitomized by the English mathematician and philosopher Alfred North Whitehead, it offers another way of thinking about “stuff” —what if reality is not made of substances but of processes, the world not made of things but of events?

At first, this idea defies not only how we see the world (still appearing to be made of solid objects), but also how we think we can possibly conceive the world. First, like fish not realizing they are constantly swimming in the water, we have been conditioned to think like this throughout our lives. Second, at the civilization level, Western thought has championed an “ontology of stones” for centuries (other traditions, however, illustrate and demonstrate that other valid systems of thought are possible). Third, as a species, stones have indeed always looked very real helping us to hunt and ultimately survive (but so has fire). It is somewhat irresistible to consider stability as more fundamental than change.

Apart from the experimental findings and theoretical realizations of physics, the notion of an “object” involves several fundamental difficulties. The perennial problem of change (the famous Heraclitean claim that it is not possible to step twice in the same river) challenges the very notion of identity. You change, and yet you are still you. But even more: your skin, your hair, and virtually everything in your body is soon ultimately replaced. Similarly, one may ponder: how many pieces can we remove from a car until we no longer consider it a car? Or, how many hay stalks does one need in order to have a haystack? Under the substance paradigm, despite positing enduring essences, change and identity seem incongruous. Things are what they are, and yet they change all the time. How to reconcile the two?

The idea of identity has not ceased to obsess the modern imagination. After physicists went after it by decomposing matter, biologists, imitating the model of physics, set themselves the same agenda (notably, and ironically, while physics itself was realizing its futility): to study living organisms by breaking them into tissues, tissues into cells, and cells into molecules. They did not go further since once one dives inside the molecule, quantum physics changes the game. Carried away by a kind of architectural metaphor they thought that decomposing things into their fundamental elements would reveal the “bricks of the real,” all simple, all identical.

However, the intellectual boldness of physicists taught us that when you get to the smallest bits, not only doesn't the universe look like a uniform pile of bricks, but that such a zoo of particles within exotic families (quarks, leptons, gauge bosons, etc.) are not localizable or distinguishable them from the field in which they move, and from which they appear and disappear. Activity was not a by-product of stability anymore, but the other way around. The elemental was conceived as an expression of the perturbed. Substances, upon close inspection, turned out to be stabilized processes. The foundations upon with Western thought is built were literally and metaphorically shaken about a century ago.

And yet, for any formulation or adoption of a cosmological theory, it seemed necessary to postulate a continuous matter with permanent attributes that persists and retains its identity over time, a matter that changes but is numerically identical to itself and maintains its *identity* despite all accidents and transformations. This idea has shaped the basis of scientific materialism for the last centuries. We can recall the scientific formulation of activities associated with empty space that, in the nineteenth century, produced the materialistic ether as the substratum of all transformations and changes.

But one does not need to ponder the ethereal. In *Process and Reality*, Whitehead uses the example of a stone. Today we conceive the stone as a set of separate molecules in continuous agitation: “But the metaphysical concepts, which had their origin in a mistake about the stone, were now applied to the individual molecules. Each atom was still a stuff which retained its self-identity and its essential attributes in any portion of time—however short, and however long—provided that it did not perish. The notion of the undifferentiated endurance of substances with essential attributes and with accidental adventures was still applied” (Whitehead, 1929, p. 78). According to the English philosopher, this is the substantialist foundation of materialism. Matter becomes a metaphysical concept, a final reality, imperceptible, and that exists regardless of its qualities, regardless of our own observations. Such “stone ontology,” as Whitehead justifiably claims, has shifted from the stone to the particle. And then from the particle to everything else.

The Cartesian conception of reality—upon which the majority of sciences are still based—is one of “bricks and mortar,” atoms and their *interactions*. It is important to realize that the mortar does not change the brick in any way, but just its external relationship with other bricks in space. A brick remains a brick, regardless of all the other bricks. Each brick of reality has a place, where no other brick can be.

This is what, according to Locke, gives each brick its identity. Bricks are what they are by virtue of their instantaneous being just where they are and nowhere else. Locke's *principium individuationis* states that “the only thing which differentiates one atom from all others is its spatial location at a certain particular instant and nothing else” (Locke, 1689, II:XXVII). Differences are thus only differences in spatial location. This entails the possibility to endow a “definite portion of space with well-defined boundaries.” Modes of thought based on a substance ontology thus easily lend themselves to materialism, reductionism and mechanicism: the world is made of (and reducible to) building blocks, which are all physical, each occupying a different place in space. Being external to one another, their identities are, in essence, independent. It is their spatiotemporal location that grants them their identity.

We are also led to think that at the bottom such bricks are identical, since what makes them different is only where they are. Note that not only can we hardly conceive what an electron really is, but we are convinced that there is such thing as two identical electrons. Leaving aside Whitehead's puzzling remark [“an electron within a living body is different from an electron outside it, by reason of the plan of the body” (Whitehead, 1925, p. 79)], the fact is that it is not possible to delineate any such entity. We do not know where an electron starts nor where it ends. They are expressions of activity in a field. Their localization would in turn become problematic.

This habit of the intellect also applies to macroscopic objects. We see a cat running after a mouse. Despite their interaction, the cat and the mouse are deemed to be distinct and separate. According to this worldview, all things are conceived as having modes of existence that (no matter how much one wishes to emphasize their interactions) are fundamentally separate. But, is an essentially disconnected universe still a universe? How to avoid such a fundamental separation?

Even if one supplements such a worldview with the possibility of every bit of stuff to act on every other bit, such action is nothing more than displacement in space (A pushes B). Thus, in a world made of particles, their relationship occurs via *inter-actions*. Interactions are mechanical insofar as the only change that they allow is rearrangement. All change is due to the displacement of discontinuous, rigid, compact units guided by mechanical laws. Such units are what they are, and will remain what they are, by virtue only of themselves: located in space and unchanging in time.

In such a world, differences in kind must be apparent. The spatial configuration of the elements can change; their inner natures cannot. There is not only separation between objects, but also within them when it comes to their qualities. A classic example that both illustrates and defies this point is that of the cloud, yellow at dawn, white at noon and pink at sunset. Color would not be something inherent to the cloud because it changes as the light changes. Since Locke, the idea that color was inherent in things was abandoned. The object, well defined, had been separated from its color (and from the subject that perceives it).

Moreover, such a universe would “read Braille” since the only way to know of each other would be by direct contact, touch, impact. The universe is then conceived as a cosmic billiard board of *simply-located particles* whereby each bit of matter would, by definition, be individually independent, “regarded as fully describable, apart from any reference to any other portion of matter” (Santos and Sia, 2007, p. 91). Ironically, relations are simultaneously deprecated and deemed necessary to glue the world together. In a world made of *externally-related “stuff,”* any relation to another entity is always secondary, if not counterfeit.

The primary aim of this article is to make explicit the pervasive commitment to “simple location” and to articulate its pernicious consequences. Such a negative critique is positively supplemented with an alternative, based on Whitehead's idea of “internal relations.” The outline of the article follows this logic and then qualifies the discussion about simple location in the context of scientific and metaphysical abstractions by explicating the so-called “fallacy of misplaced concreteness.” Then we address “process thought” more widely, briefly discussing its origins, current flavors and subtle caveats, especially with respect to dynamical systems theory. To make those ideas more concrete, we provide several examples of the power of process thought across disciplines, with an emphasis on the cognitive sciences. We end with an outlook on the prospects of conceiving *inter*-identities as *intra*-identities, thus transcending reductionistic and mechanistic stances, even when still covert in certain organic and processual views of matter, life and mind.

Ultimately, and more generally, the conceptual challenge entailed by our proposal is *to think change without vehicle and container*, namely, to oppose “the idea of an inert, unchanging container of physical becoming” filled with physical particles and based on “relations of mutual exteriority which are characteristic of classical space” (Capek, 1971, p. 271). In other words, to abandon the idea that motion is *of something* (matter) *in something* (space), both considered in timelessness, which is nothing but an abstraction of concrete reality. Put differently, the problem with dualism –the idea that there are two substances, body and spirit– is not so much with the word “two” but with the word “substances.”

## Simple Location

After such an introductory detour, we are now in position to ask: What is the foundational assumption upon which the above notion of identity rests and which, at the same time, creates so many theoretical problems? Whitehead argues that it is a conception he calls *simple location*: “By simple location I mean one major characteristic which refers equally both to space and to time (.). The characteristic common both to space and time is that material can be said to be *here* in space and *here* in time, or *here* in space-time, in a perfectly definite sense which does not require for its explanation any reference to other regions of space-time. (.) and, so far as simple location is concerned, there is nothing more to be said on the subject” (Whitehead, 1925, p. 49).

Thus, *simple location* is the notion that there are portions of matter that are fully describable apart from any reference to any other portion of matter, so that any relation to other entities, existing or not, is secondary. Relations thus cannot really say anything about the internal constitution of a bit of matter. When it comes to space, this entails the possibility of completely isolated systems (e.g., the so-called “brain in a vat”). For time, it means that change is sequential rather than serial, and that duration can be shrunk to an instant. These aspects imply a fundamentally disconnected universe in space and in time.

While the realization of the impossibility of a completely isolated system may indeed trigger a conversion to a relational view of physics (Smolin and Mangabeira Unger, 2014), thinking about relationality can still miss the key distinction between external and internal relations. Thus, the acceptance of *simple location* is what needs to be criticized at the core, as “[t]his idea is the very foundation of the seventeenth century scheme of nature” (Whitehead, 1925, p. 58).

Once simple location is assumed, several scientific and philosophical problems follow: how to conceive memory, causation, induction, evolution, ethics? In an entity externally related to itself in time, the past cannot enter into the present. Again, by which procedure can it be linked back? If we take simple location seriously, the movement of a particle becomes impossible. Simple location causes serious problems to induction as well. If each configuration of matter has no inherent references to any other place or time—if nature is really like this, external to herself—then induction is not based on anything inherent in nature; “the notion of ‘simple location' is inconsistent with any admission of ‘repetition”' (Whitehead, 1929, p. 137); the consequences that Hume pointed out were correct, had his premises been true. Furthermore, external relations do not allow for evolution. If one is to have something else than mere *unfurling* (Gomez-Marin, 2020), a doctrine of *internal relations* is necessary: “The aboriginal stuff, or material, from which a materialistic philosophy starts is incapable of evolution. (…) There is nothing to evolve, because one set of external relations is as good as any other set of external relations. There can merely be change, purposeless and unprogressive. But the whole point of the modem doctrine is the evolution of the complex organisms from antecedent states of less complex organisms” (Whitehead, 1925, p. 107). In order to allow for personal development and ethics, simple location must also be rejected. Identity, as the quality of being the same to oneself, leads to the following situation: A may interact with B, and some properties of A may even be affected, but A will remain equal to itself regardless of B. If things—by definition externally related—are the most fully real, and enduring things are self-identical through time, then no true development can occur. In addition, an ethics in which your relationship with others is fundamentally different than with yourself seems doomed to fail.

One may trust that by supplementing the parts with dynamic interactions one can ameliorate the situation. But emphasizing interactions of otherwise simply located elements does not bring forth a more internally related universe. To put it metaphorically, the taint of simple location cannot be cleansed by rubbing; we submit that the cloth must be abandoned. The problem of interactions is itself problematic. The adoption of simple location is a major drawback to the reasonable “fix” of emergent properties.

In fact, contemporary versions of emergentism seek to correct reductionism with the help of mereology. This is certainly important, as we need to be able to distinguish between different senses of “parthood.” How the parts relate to the whole is what is at stake. Is the whole prior to its parts? If so, one must ponder where it is so logically, chronologically and/or ontologically. Commendable efforts to reject reductionism in favor of holism still adhere to materialism (Gilbert and Sarkar, 2000), perhaps unable or unwilling to reject the commitment to *simple location*.

It is instructive to revisit the concept of mass as an example of how “holistic” narratives can still carry out the baggage of the notion of simple location. “Newton defined it as *vis insita*, that is, literally, as *force residing* within the location occupied by matter and constituting, so to speak, its substantial nucleus which is related *externally* to other particles. The belief in the simple location of sharply defined corpuscular entities could have hardly found more accurate formulation: the essence of material particle is its resistance to acceleration, reacting *hinc et nunc* against the *external* influences of other equally well defined corpuscular entities” (Capek, 1991, p. 209). Or, quoting physicist and philosopher Ernst Mach's criticism of Newton: in the principle of inertia there is “an abbreviated reference to the entire universe” and that “the neglecting of the rest of the world is *impossible*” (Capek, 1991, p. 210).

The critique extends also to conceptions of interactions in physics: “to isolate one particle and force from the whole dynamical context is as artificial as to claim that buying may take place without selling” (Capek, 1991, p. 210). Maxwell realized that Newton's third law unifies action and reaction as one dynamical phenomenon: *stress*. Action and reaction are two opposite effects of the same reality, in the same way that in “commercial affairs the same transaction between two parties is called Buying when we consider one party, Selling when we consider the other, and Trade when we take both parties into consideration” (Maxwell, 1992, p. 27). For Faraday, “matter is not merely mutually penetrable, but each atom extends, so to say, throughout the whole of the solar system, yet always retaining its center of force” (Capek, 1991, p. 178).

After Faraday and Maxwell, modern physics irreversibly stumbled upon the problems that *simple location* creates. In fact, a century ago such a concept was left virtually unrecognizable after Relativity Theory and Quantum Theory. Due to the principle of indeterminacy and entanglement, precise boundaries became ill-defined and particles could not be localized anymore.

Inspired and spurred by the radical worldview transformation afforded by modern physics, Whitehead denied the concreteness of simple location. He did not prune it; he pulled it out from its root. Our goal here is to be able to think in an intrinsically relational manner by means of Whitehead's *event*-notion of individuality and his doctrine of *internal relations*.

## Internal Relations

The negation of *simple location* is accompanied by an affirmation. Whitehead puts forth the notion of *internal relations*^1^, which he introduces when discussing Einstein's relativity. Space-time relationships have been generally understood as external relationships. Whitehead denies that. He resembles Leibniz when he states that the relations that an event has are all internal relations: “This internal relatedness is the reason why an event can be found only just where it is and how it is, that is to say, in just one definite set of relationships. For each relationship enters into the essence of the event; so that, apart from that relationship, the event would not be itself. This is what is meant by the very notion of internal relations. It has been usual, indeed, universal, to hold that spatio-temporal relationships are external. This doctrine is what is here denied” (Whitehead, 1925, pp. 122–123). Put plainly, an internal relation is a relation between entities such that it is not possible for them to exist without each other. Thus, from the stance of the doctrine of internal-relations, inter-actions are “add-ons” to substances; a glue between “things” which, in turn, do not need the glue for their being.

Internal relations determine the identity of the related entities. For the purpose of gaining some intuition about this notion, let us provide some examples within a somewhat heterogeneous list of cases. A mother and her baby (specially a fetus) can be said to be internally related because their mode of relation implies that one could not properly speak about the latter if one would leave the former out. In other words, they owe to each other what they are. Another case of entities whose existence is intrinsically relational is that of a bee and its hive. So is quantum entanglement, where two physical systems, despite not being in interaction at the present time, are inseparable beyond accounts based on shared memory or the common cause principle. In the realm of cognitive sciences a curious example is the gathering of a magic trick, since the magician cannot do magic without a spectator (it is easy to fool oneself, but it is impossible to do a magic trick to oneself). Escher's Drawing Hands may serve as a visual analogy to grasp internal relations.

In sum, that the properties of A depend on B causes no theoretical problems. But claiming that the identity of A depends on B defies the intellect. It is true that one can conceive of things in external relation and still claim that it is impossible, for some of them, to exist without the other. The real challenge is to conceive a mode of relation that determines not only the possibility of existence but the essence and identity of two “things.” Process philosophy—at least for Whitehead, as we are trying to explicate here—undertakes such a task.

But, if things are not really where they are, does this mean that they are everywhere? Whitehead claims: “In a certain sense, everything is everywhere at all times. For every location involves an aspect of itself in every other location. Thus every spatio-temporal standpoint mirrors the world” (Whitehead, 1925, p. 91). At first, this may seem a disproportionate claim. Whitehead's proposal is not so much to claim that a particle is everywhere but that, in a precise sense, it also can and must be where it is not. The critique of simple location implies the negation of well-defined regions in space and time. Events are spread out (and, importantly, they also have a temporal width). Their boundaries are fuzzy. Upon inspection, objects are not everywhere in the same sense, but they do indeed enter into the beings of other entities, and this way of being in others is what constitutes a thing's location. The world is made of entities that are here and also, in a way, somewhere else. Whitehead's theory involves the complete abandonment of the notion of simple location as the way in which things (or, more precisely, events) are in space-time.

In closely examining his critique of simple location, Capek qualifies Whitehead's “mirroring the universe” by means of emphasizing the causal cone of events: “each particular event reflects that part of the universe which acts on it as well as the potentialities of its own future effects; *but it remains causally unrelated to those events which neither act on it nor will be acted upon by it”* (Capek, 1991, p. 215). Thus, although events are not simply located, they are circumscribed to causal influences. This supplements the principle of internal relations by limiting the repudiation of simple location. In other words, while one can still say that “each particular event mirrors the world,” what is meant by the word “world” is not a complete entity outside of time, since “the act of mirroring *takes time*, that it is itself a time-consuming process” (Capek, 1991, p. 213).

Paradoxically, simple location seems to adequately reflect experience (but it does not). In one way we see objects “out there,” simply located; simple location would then be a mere transcript of the obvious facts. But, on the other hand, experience cannot confirm simple location for us as an elemental fact (it is an abstraction). Whitehead insists that to try to understand his proposal in terms of our everyday notions of time and space will inevitably bring great paradoxes. In contrast, “if you think of it in terms of our naive experience, it is a mere transcript of the obvious facts” (Whitehead, 1925, pp. 91–92). There is no element apprehendable in immediate experience were simple location is to be found. And yet, a paradigmatic rebuttal reads: “if our experience shows the contrary, so much worse for experience!” (Capek, 1991, p. 205).

## Misplaced Concreteness

The problem with simple location is not just the simply attributed location by itself. It is that we take such an *abstraction* as *concrete*. In other words, the error is to conflate abstraction with reality; what Whitehead called *the fallacy of misplaced concreteness*.

Are we denying the reality of atoms? Yes and no. Atoms are both invented and discovered. No one has ever directly seen one. And yet, there is empirical evidence for them. However, upon close inspection, their essential properties crumble, as could have been expected^2^. Atoms turned out not to be atomic. While they may still be useful abstractions, the problem is to forget that “atomicity is only one aspect of nature” (Capek, 1991, p. 198). Let us go back to Maxwell and quote him at length: “We are accustomed to consider the universe as made of parts, and mathematicians usually being by considering a single particle, and conceiving its relation to another particle, and so on. (.) To conceive a particle, requires a process of abstraction since all our perceptions are related to extended bodies, so that the idea of *all* that is present in our consciousness is perhaps as primitive an idea as that of any individual thing. Hence there may be a mathematical method in which we proceed from the whole to the parts instead of from the parts to the whole” (Capek, 1991, p. 179). Upon abstraction, the intellect assumes not only that things are isolatable in our mind but also that they are isolated in reality. Put plainly, that despite their not being isolatable, when we do isolate them, they do not change in kind. We conflate useful ideas as fundamental statements about the world.

Abstractions are indeed useful (actually, this is their *raison d'etre*). We are constantly abstracting in our daily life. If we want to take a train, we abstract from the train only that which is of our interest: schedule, price, destination. We do not attend to the color of the upholstery, the decoration of the toilets, or where the engine was made. We do the same in our personal relations. The advantage of abstractions is to limit thought to things and relations that are clearly defined (clarity is often at odds with precision). Thus, the error does not lie in making abstractions, but in taking them as concrete. In sum, abstraction is paradoxical in that its utility depends on its falsity. If what abstraction excludes is important to experience, then this mode of thought becomes inadequate.

Let us say it more clearly: no abstraction, no thought. And without thought, there is no science. However, it is also true that: no concreteness, no life. We must abstract from the world in order to think about it, but we must also attend to the concrete particulars in order to live in it. So it is not possible to do science without abstraction, while at the same time it is possible to grasp the concrete by means of our immediate experience. If we are incapable of questioning—and eventually getting rid; or at least temporarily suspending—of our familiar abstractions, our work is condemned to sterility. As a group (scientific, or otherwise) we would literally live auto-enclosed and un-grounded. In this sense, the role that the philosopher can play as the critic of abstractions becomes decisive for science.

So, if one never really lays hold of the “thing in itself,” if science must abstract in order to study the concrete, how to tell whether one abstraction is better than another? Exactitude depends on our interests: “What I am opposed to is the concept of some ideal exactitude given us a priori, as it were. At different times we have different ideals of exactitude; and none of them is supreme” (Wittgenstein, 1980, p. 37). Abstractions are subordinated to our interests, intentions, desires, and values (which are always human values). Accordingly, a separation between the sciences and the humanities is not possible. In fact, science would be nested in the humanities, rather than the latter being a sprout subordinated to the former.

Science is exact, its predictions can be tested and the whole enterprise is, above all, useful. And yet, “[i]t turns out that physical truths, upon their theoretical qualities, had also the condition of being profitable for the vital conveniences of men. Starting from those, men could intervene in nature and make it comfortable in their own benefit” (Ortega y Gasset, 2015, p. 272; our translation). Thus, scientism can be defended by the bourgeoisie, since “comfort is simple a subjective predilection (.) but one that does not reveal by itself any superiority of character” (Ortega y Gasset, 2015, p. 272). The criterium of utility need not supersede that of truth, or any other. If what science does is indisputable, what it says about what it does must be disputed (Canales, 2015).

In consequence, and contrary to Feynman's noted dictum, we contend that philosophy of science is as useful to scientists as air is to birds. There is always a metaphysics at work (and believing there is none is the most dangerous kind of metaphysics). Process thought offers a viable alternative to “bricks and mortar.”

## Process Thought

Process thought is not a novel invention of the twentieth century, as it represents the continuation of a tradition that started with Heraclitus, all the way to Leibniz and Schelling, amongst other philosophers. Bergson can be considered its forerunner at the beginning of the last century, acknowledging William James as well [he wrote: “What really exists is not things made, but things in the making” (James, 1909)]. Whitehead drew from both Bergson and James. As he himself acknowledged, “I am also greatly indebted to Bergson, William James, and John Dewey. One of my preoccupations has been to rescue their type of thought from the charge of anti-intellectualism, which rightly or wrongly has been associated with it” (Whitehead, 1929, preface). Whitehead had unique credentials to also address the mathematical and physical aspects of process philosophy in the context of modern science. Not widely known (and at times ignored), Whitehead is nevertheless arguably the major exponent of process thought.

Despite his rather humbling remark that all of Western philosophy is a series of footnotes to Plato, Whitehead embarked on a challenge that, to our knowledge, no other philosopher has achieved, nor probably sought, namely, to integrate the three apparently incommensurate worlds of clocks, quanta, and consciousness. Thus, he provided a coherent account of the familiar classical behavior of macroscopic objects, physical theories of the time on the ultimate entities of matter, and the world of subjective experience. Whitehead's philosophy is not only an ontology but also a cosmology. Its attempted scope is the entire cosmos.

To that ambitious end, in his *magnum opus* Process and Reality (Whitehead, 1929) he introduced a set of complex new categories and axioms, which constitute his unique philosophical scheme. This makes his philosophy quite impenetrable at first reading (and at second and even third readings as well). It is thus not possible for us to unpack Whitehead's complex metaphysical scheme in this manuscript (nor is this our purpose). In fact, this is the reason why we set ourselves the modest but still daunting task of introducing and conveying three of its core elements: the critique of simple location, the fallacy of misplaced concreteness, and the doctrine of internal relations (whose discussion, by the way, appears in his much more accessible book, Science and the Modern World). In our view, these three ideas are fundamental to be able to think inter-actions at the physical, biological and mental levels in a way that does not, explicitly or implicitly, assume a mechanistic worldview whereby relations may be deemed important but ultimately not essential.

Whitehead's process philosophy rejects any actuality that is static in order to affirm that all actuality is processual (Cobb and Griffin, 1976). In a nutshell, his “philosophy of organism” consists in replacing substances with events as the entities that make up the real. As we have argued above, objects are entities that are external to one another, forming systems of parts that are fixed despite their interactions, no matter how much one emphasizes their relations. Events, in contrast, are conceived as persisting processes. Whitehead provides a whole new metaphysics that departs from the notion of substance.

The difficulty of Whitehead's process philosophy has been met with a poor reception. Some scholars admit that “its detractors, principally from the new analytical tradition (…) consider Whitehead's most recent work obscure, confused, wooly and mystical, not worth the effort of reading or trying to understand” (Simons, 2013). It is indeed challenging to think in a process manner if one speaks a “substance language” (where nouns sound like substances). Whitehead coined new words and used existing ones with different specific meanings precisely in order to create a universe of discourse that could bypass “substance thought.” According to Isabelle Stengers, Process and Reality “is a text whose obscurity has put off many readers but which I wish to defend against a particular way of being read.” She adds: “you cannot read Process and Reality from the first to the last page, in a linear manner, but must zigzag, using the index, being lured to come back to something you recollect but which had remained mute and now takes on a new importance, taking the leap that you have just felt is possible” (Stengers, 2008). Indeed, many have given up.

Paradigmatically, prominent members of a recent revival of process philosophy for biology are “more inclined to risk reinventing the wheel than to look for the concepts and theses we want in Whitehead's metaphysical system” (Nicholson and Dupre, 2018, p. 7). In contrast, here we wish to keep the rider and the horse. We sympathize with the current efforts, especially in theoretical biology, that stress that “what is alive is not really a thing.” However, an adequate diagnosis of the limitations of a substance ontology does not guarantee an accurate prognosis. Namely, Whitehead “understood that to make all permanence illusory, to deny being in the name of becoming, to reject entities in favor of a continuous and ever-changing flux meant falling once again into the trap always lying in wait for philosophy.” (Prigogine and Stengers, 1984, p. 89). Ultimately, reiterating that everything is a process does not do the explanatory work.

Moreover, the view that process equals change (which, in turn, can be properly captured by dynamical systems) is misguided and naïve. Repeating the “everything flows” mantra may increase the popularity of process philosophy amongst biologists (Nicholson and Dupre, 2018). One must be wary not to do so at the expense of its precision. As we will argue next, one may end up with a surrogate version more akin to dynamical systems theory spuriously upgraded to a kind of process ontology. Again, while emphatic narrations of the processual nature of reality are welcome and needed (Jaeger and Monk, 2015), the processual virtues of dynamical systems modeling may break down when one examines whether they can accommodate internal relations. We submit that there are important “performative contradictions” whereby what is claimed contradicts what is assumed. To put it plainly, one can emphasize dynamic interconnectedness in order to defend a process-based view of nature while still (perhaps unknowingly) embracing a substance-based view. Having said that, let us make clear that one does not need to be a Whitehead devotee to cultivate process thought.

Even in the context of our critique of simple location, it can seem rather futile to point to the inadequacy of dynamical systems theory. After all, its great effectiveness as a mathematical formalization to study the behavior the physical world is more than attested (moreover, what comparable practical alternative have we got?). Stemming from classical mechanics, and conceived to describe the movement of projectiles, planets and falling apples, it is arguably the mathematical formalism *par excellence* to model not just the behavior of inert matter, but also of life and mind.

That cognitive agents are (more like) dynamical systems rather than digital computers (van Gelder, 1995) can be seen as an upgrade on the cognitivist metaphor, too often ingrained to the point of dogmatism. Researchers across disciplines seem perfectly fine lending to differential equations (or to difference equations, when time is treated discretely) the job of mathematically modeling development, evolution, or cognition. In the words of Evan Thompson, “[a]ccording to the enactive approach, the human mind emerges from self-organizing processes that tightly interconnect the brain, body and environment at multiple levels” (Thompson, 2010, p. 37). Autonomy is particularly underscored. Self-determining systems (autonomous systems) are not completely determined from the outside (as in heteronomous systems). The interactions of autonomous systems with the environment are thus more akin to “conversation” than to “commands” (Pask, 1980). As long as interactions, emergence and autonomy are emphasized, it seems that one can safely borrow the dynamical systems formalism. But, can we?

There is no doubt that nature is “dynamic” (this is, characterized by change, activity, forces and movement). *Lato sensu*, dynamical systems are collections of interrelated entities (“system”) that change over time (“dynamical”). In essence, in mathematical dynamical systems a function describes (or prescribes) the temporal evolution, deterministic or stochastic, of a point in a geometrical manifold that is called phase space. We should take dynamical systems seriously, but not literally. As mathematical abstractions they can be confounded with the concrete actual entities they seek to represent. However, we argue that there is more to this than just the well-known warning that the map is not the territory.

Are organisms *really* dynamical systems? The process philosopher Spyridon Koutroufinis has dealt extensively with this problem (Koutroufinis, 2014b, Koutroufinis, 2017). A whirlpool may very well be a better model for a fruit fly than a clock. But, in fruit flies, not only their trajectories but also the dimensions in which they unfold emerge along with the processes. In contrast, dynamical systems are generated in a *phase space*, which represents all the possible states of a system. Note how, unlike living organisms, the possible states of a dynamic system are defined independently of the agents in it. There is no co-evolution of the agent and the phase space. Nor is the principle of change inherent to the agent. In addition, such space is fixed. Furthermore, its axes imply that the concrete is made out of a combination of universal generalities. Finally, the trajectory is a succession of immobilities. There is flux, but it supervenes (it is not essential). In fact, movement in a space of a given dimension can be recast as a static shape in another space to which one adds one more dimension (time is “spatialized,” as Bergson incessantly denounced). Equations express accomplished or accomplishable facts rather than facts in the making. As opposed to the reality they portray, such mathematical tools do not endure. In sum, the main shortcoming of the dynamical frame for process philosophy is that one gets a proxy of Heracletean change within a Parmenidean space.

A key difference between machines and organisms is that, in the former, constraints are imposed from the outside while, in the latter, they are imposed also from the inside. One thing is to stress the constitutive reciprocity of an agent with its environment, another to grant that organisms determine the relevance of their environment not only by means of what they take of it but also by actually crafting it. We do not mean that relations determine what an organism is, but that the organism determines itself through its relations to its *Umwelt* (von Uexküll, 1992). The former position would imply a sort of radical relationalism, a reduction of the subject to its relations. The latter is a process ontology whereby the experiencing subject is essential because it is a center of action in the world. In turn, the question of subjectivity begets the question of the environment. So, in order to properly answer “Is cognition in the head or in the world?,” one must ponder and clarify: what world? (Feiten, 2020). Physical surroundings and meaningful environments are different worlds. Even if one rejects “computation” (representation, as in cognitivism), and puts forth “affordance” (selection, as in ecological psychology) or “enaction” (construction, as in enactivism), accounting for subjective experience remains a challenge.

Furthermore, in the framework of dynamical systems there is no room for spontaneity, creativity or the appearance of true novelty; something happens only because something else makes it happen (adding stochasticity does not change the picture). Even when committed to interactions, emergence, and mutual dependencies, there may be no room for self-determination. In contrast, Whitehead's process ontology postulates that the world is made of events or processes that are not only interwoven, but also creative. Whitehead “understood perhaps more sharply than anyone else that the creative evolution of nature could never be conceived if the elements composing it were defined as permanent, individual entities that maintained their identity throughout all changes and interactions” (Prigogine and Stengers, 1984, p. 89).

Therefore, each process (and, for that matter, every individual) is then understood as a developing subject not completely brought about by efficient causes. To radical relatedness we add intentions of our own. As Koutroufinis remarks, “the prehended facts of the past do not push the process into the future in the way in which the causality of classical physics does (…). The present is not the passive and trivial transition from a complete past into a predetermined future. This is because the process decides, in its present, which factors from the past are to be considered relevant and which role the selected factors will have in forming the future” (Koutroufinis, 2014a, p. 19).

It is revealing (and somewhat amusing) to realize that one has never seen an equation that changed itself. The rules are fixed. Dynamical systems change, but the laws that govern them do not. When dynamical systems are further abstracted from the equations themselves to Hamiltonian and Langrangian mechanics, and then further into symmetry principles (à la Noether) one can begin to grapple with the origin of such fundamental limitations: being two of the foundational theoretical aspects of physics, fixed phase spaces and symmetries (with their associated invariants and conservation laws) do not to lend themselves well to biology (Longo and Montévil, 2012).

We cannot add a concrete way forward at this point, except from articulating these fascinating challenges. In any case, the absence of a solution does not make a problem disappear. There are some silver linings, though. Inspired by Deleuze and Guattari's notion of “differential heterogenesis,” and in line of Simondon's concept of “individuation,” a mathematical framework for “heterogenetic becoming” has been recently proposed where constraints can themselves change in time (Sarti et al., 2019). In contrast with mathematical physics (which would be a form of “symmetrization of heterogenesis”), the morphogenetical space is not given a priori, namely, there is morphogenesis *in* space but also morphogenesis *of* space. Despite currently under construction, new special mathematics seem to come to rescue us from this situation. Let us bear in mind that mathematics is also a human historical endeavor (it is what people make of it). Ironically, it may turn out that in our celebrated attempts to naturalize physics we may have actually physicalized nature. It is perhaps time to nest physics in biology with the help of new mathematics informed by philosophical thinking (Longo, 2020).

Going back to the notion of internal relations, once more the question is not settled by emphasizing the processual nature of whirlpools (or, to that matter, the processual nature of the toilet in which whirlpools form when we flush it). In any dynamical system there is always a final level of internal relationship below which basal elements (entities or variables) are defined independently of their relations. Such mathematical theories of dynamical behavior, operating under the implicit assumption that basal elements have a fixed essence, presuppose the externality of the relations of the basal elements. Even if one claims that interactions are more important than their constituents, this claim holds only for the behavior of the system, not for the constituents themselves. “It is arguably this inseparable connection between processuality and internal relationship that also creates the biggest difficulty in Whitehead ontology: The process which gives rise to relations of experience exists prior to them neither logically nor temporally. The processual subject only comes into being through its relations with other subjects.” (Koutroufinis, 2014a, p. 16). In a word, no internal relations, no process.

To that end, we may distinguish a weak and a strong sense of process. Equating process with change entails a “light process philosophy.” In the non-philosophical usage of language this happens most of the time. Even Whitehead talks about the concrescence process (which is the actualization of an actual entity) and the transition process (which is the transition from one concrescence to the next one). Whereas “concrescence” denotes actualization and self-determination, “transition” may be understood as nothing more than change of position within classical mechanics, as in the movement of a car, a ball, or a planet. However, transitions consist of processes. Only a view from the distance gives the impression that the transition is a mechanical movement. In contrast, in our view, process in a strong sense (à la Whitehead or Bergson) is a becoming that determines its own aim through its own actualization. In order to do so, it specifies its relations to the facts of the world. It is the self-determining entity that decides what role the facts of the world will play in its self-determination. It is a self-determining experiential act embedded in an Umwelt.

Let us reiterate that we are not demanding organicists to embrace Whitehead's philosophy of organism. We are calling attention to the metaphysics that operates underneath one's theory (which in turns frames the data we collect and how we interpret it). However, some researchers may claim that they have no philosophical commitments, or that one's philosophical stance is irrelevant to science. In the worst-case scenario, a *covert* substance ontology lies at the bottom.

## Applications Across Disciplines

Whitehead's thought has concrete implications and finds specific applications to a wide range of important questions. Not only is it more inclusive of the evidence but, under its lens, many disciplines become integrated, namely, they cease to be “externally related.” Let us illustrate several key cases:

First, Whitehead's metaphysics provides a philosophical basis to the universal experience that “nothing lasts,” without overstressing it to a point where everything would simply be pure change. As John Dewey wrote: “The modern Heraclitean is Alfred North Whitehead, but he is Heraclitus with a change. The doctrine of the latter, while it held that all things flow like a river and that change is so continuous that a man cannot step into the same river even once (since it changes as he steps), nevertheless also held that there is a fixed order which controls the ebb and flow of the universal tide” (Dewey, 1998, p. 219).

Second, Whitehead's philosophy is coherent with quantum mechanics (Epperson, 2004) when it comes to articulating a philosophy that can affirm, without self-contradiction or cumbersome contortions, that “there is no nature at an instance” (including non-locality in space). That is, time without duration is an abstraction. Note that, before Whitehead, the paradigm changes of modern physics had not taken place yet. After him, and despite the fact that most of logical positivism was motivated by the change of view brought about by Einstein's relativity theory, very few philosophers had Whitehead's mastery of mathematics allowing to integrate such advances into their philosophical schemes. A process conception of reality takes temporality seriously. Creativity takes time. There is process all the way down.

Third, Whitehead's organic doctrine entirely resonates with the claim that “nothing exists in itself.” Process thought entails a radical ecological position. Ecology here is not to be understood as recycling plastic bags but as giving primacy to a relational conception of nature, rejecting classical physics and neo-liberal economics as the foundations of the natural and social sciences, respectively. Process thought provides the philosophical foundations of an ecological civilization and poses urgent corrections to the course of our human ways of life (Vltchek and Cobb, 2019).

Fourth, process philosophy has allowed great advances in theological thought [one may wonder who cares about theology, until realizing that scientific efforts to get rid of religion still carry deep rooted in their fundamental axioms a whole range of theological commitments; see for instance (Riskin, 2016)]. Process theology has dared to reject a fundamental pillar of traditional (substance-based) theology: the doctrine of an omnipotent creator. Under a Whitehedean perspective, God is not synonymous with “the Almighty” (it is not necessary to explain here what exactly Whitehead means by God, but it is certainly not the old-bearded man watching us from the sky). The “cosmic community,” as Barbour puts it, “is neither a monarchy nor a democracy, since one member is preeminent but not all-powerful” (Barbour, 1991, p. 3401). Beyond academic armchair corrections, this resolves a question that theology always struggled with, namely, how come there is evil in the world (Cobb and Griffin, 1976). Moreover, praying can then be conceived as an attempt to persuade God, rather than mere psychological talking-to-oneself (or, as Kierkegaard would say, “to change the nature of the one who prays”). Finally, and irrespectively of any particular religion or ethics, personal responsibility has a natural place in Whitehead's philosophy. Moral effort is real and meaningful.

Fifth, when it comes to theoretical psychology, how does Whitehead's philosophy apply to the study of the human psyche? To our knowledge, process thought has had fewer incursions in psychology than in other disciplines. A process-oriented conception of the human being has been discussed in the context of psychiatry (Koutroufinis, 2002) and psychotherapty (Cobb, 2000). In turn, an affect-based account of human experience and emotion can be extrapolated from Whitehead's critique of pure feeling (Shaviro, 2009).

Sixth, in neuroscience, scarce but valuable explicit connections have been established between Whitehead and neurons. Building on a neuro-ecological model of the brain (Northoff, 2016a), a process-based ontological characterization of the brain has then been proposed (Northoff, 2016b), allowing for a (brain-based but not brain-reductive) reformulation of the mind-body problem as a world-brain problem (Northoff, 2018).

Finally, rejecting simple location has implications in the context of the study of perception. To that end, one would need to deal with Whitehead's theory of *prehensions*. However, doing so would immediately become involved and excessively technical (since, in order to explain what a “prehension” is, one would need to explain Whitehead's “actual occasions,” which in turn requires to know about “eternal objects,” and so on). Therefore, we shall briefly mention Bergson's 1896 book, *Matter and Memory* (Bergson, 1896), where he proposed a theory of perception that can be considered a precursor of modern process thought in the realm of cognitive sciences. According to Bergson, we do not perceive the objects of sensation in our brains but in these very objects (which, in turn, reminds us of Berkley's views). In connection with Whitehead's theory of prehension, perceivers prehend the objects of their sensation by participating in them. Both Whitehead and Bergson make clear that perception is an extremely reduced image of the picture; one that emphasizes the aspects of the perceived image that are useful to the perceiver (Bergson, 1896; Whitehead, 1933). We see, hear, or smell things because we are interdependent with those perceived things. The “objects” that I perceive are those that reflect the possible action of my body upon them. Conversely, perception is a selection of the virtual action of my body on them; a solicitation of the activity of my body. Despite their differences, Bergson's and Whitehead's theories of perception and memory share fundamental processual aspects.

Let us briefly consider the enactive approach (Gallagher, 2017), as it is not immune to substance metaphysics. Proposing an alternative to cognitivism, “4E(A) Cognition” goes beyond the brain, stressing that every neural subject inhabits a body, which in turn inhabits a world that it acts upon. Minds are not neural software, nor are bodies mere vehicles. Cycles of embodiment are deemed constitutive of subjective experience. The mind-body problem is rephrased as a body-body problem (a key distinction is made between “lived body” and “living body,” which we cannot cover here). Ceasing to be localizable, the mind is “spread out” in space and time, and characterized as a process. By “process” here one usually means a set of interactions whereby the system evolves in time. However, and despite the much-needed critique of neurobiological reductionism (Fuchs, 2018), it is often unclear what ontology lies beneath such phenomenology, and whether or not it subscribes to external relations. While stressing the importance of interactions, the 4E approach can operate in the direction of stressing relations as constitutive while still rejecting internal relations. Again, it all depends on the philosophy that such approaches adopt, knowingly or not.

Let us also appraise the ecological approach to perception and action (Gibson, 1979) which, like enactivism, is related (but peripheral) to the main focus of this manuscript. A first note of caution is to avoid lumping together the ecological and the enactive views (Fultot et al., 2016; Segundo-Ortin et al., 2019). The ecological approach adopts a realist ontology, advocating for direct perception, and rejecting mental constructions (Reed, 1996; Chemero, 2011). Although such an ontology (Turvey, 1992; Stoffregen, 2003) does not need to subscribe to external relations, there is still the issue of whether it nevertheless embraces a substance metaphysics or, like Whitehead's, a process one. Things get even more intricate when one realizes that it may not even make sense to talk about a human-centric phenomenology of the ecological approach since, once species-centrism is rejected (the principles of perception and action as not being different in kind across animals) access to “alien” phenomenology would remain out of reach.

We thus suggest that Whitehead's organic realism can provide an explicit grounding to the rather undetermined ontological basis of 4E approaches (enactivists expect to be realists), while offering to the ecological approach (to its non-naïve direct realism) a concrete metaphysics that does without substances.

In sum, although there seems to be a gradual reorientation toward process thought by the mainstream heterodoxy both in science and philosophy, we voice the concern that if relations remain external, then such efforts can ultimately become obstructive. Wherever there is an attempt to move toward a relational framework, substance ontology can sneak in again and hinder progress. In a way, process thinkers neglect Whitehead at their peril. Even if one decides to reject Whitehead's proposal, spelling out the Whiteheadian consequences of different philosophical approaches to accounts of perception, action and cognition may, at least, encourage scientists and philosophers to be more explicit and precise about their commitments.

## Outlook

The main mission of this article has been to draw attention to the bias of certain pervasive and arguably pernicious abstractions. Such abstractions have their common root in the ubiquitous and covert assumption of “simple location,” which is often presented as an empirical fact. Following Whitehead, we have called this into question, namely, the habit of the intellect to believe that things are *simply-located* in space and time; the idea that the world is made of things that are simply where they are. The rejection of this worldview has major consequences. More precisely, simple location incurs in the fallacy of locating concrete particulars in definite portions of space and time. Particulars are not particles. When applied to space, simple location precludes wholeness; when referred to time, it precludes creativity. The triumph of such abstractions has however prompted some of the technological development that we now enjoy. Indeed, “[t]he world of science has always remained perfectly satisfied with its peculiar abstractions. They work, and that is sufficient for it” (Whitehead, 1925, p. 66). Yet, granting scientific engineering their achievements, one must also address their contributing to the destruction of our planet (which we will not discuss here). The argument that “they work” takes technological progress and comfort as ultimate values (a claim as indispensable as indefensible).

Still in operation today, such a scientific-philosophical framework is too narrow for modern science; “it provides none of the elements which compose the immediate psychological experience of mankind. According to that scheme, there is no reason in the nature of things why portions of material should have any physical relations to each other” (Whitehead, 1925, p. 73). Paradoxically, a great deal of twenty-first century biology and cognitive neuroscience is still based on foundational ideas of seventeenth century natural philosophy and theology.

A key to abandoning simple location is the concept of field, which resonates with the notion of internal relation. A field is the set of conditions that make the event possible. For Leibniz everything is linked, everything is full, everything is continuous. We do not know if reality is continuous or discrete, or both. The problem probably has no solution and, as in the case of the One and the Many, all the solutions have been false closures. Physics tried to solve the dilemma through the concept of field, which is conceived as the continuous distribution of a preponderant condition or magnitude, described mathematically by a gradient. The concept of field, associated with structure and correspondence, has been growing in importance in physics and this relevance is now projected to biology and the neurosciences. The field can be understood as the vital space of an organism and as the totality of the possible events from which the organism's behavior will derive. If the notion of field has become essential for inert matter, it will be even more for living matter.

To use Whitehead's example: “green is not simply at A where it is being perceived, nor is it simply at B where it is perceived as located; but it is present at A with the mode of location in B. There is no particular mystery about this. You have only got to look into a mirror and to see the image in it of some green leaves behind your back” (Whitehead, 1925, pp. 70–71). Thus, the rejection of simple location is not only the denial of the self-absorbed nature of material objects in empty space, but it literally provides a different worldview from which to conceive perception.

Symmetrically, the adoption of the doctrine of internal relations is the basis for a different worldview in which things are not “out there.” It is not by chance that Whitehead traces the critique back to Berkeley: “It is in the search for this wider basis for scientific thought that Berkeley is so important. (.) the key of the problem lies in the notion of simple location. Berkeley, in effect, criticizes this notion” (Whitehead, 1925, p. 67). Whitehead brings forward—perhaps more vigorously, but also in a more balanced way— a critique that Berkeley pioneered. The Irish philosopher questioned the existence of self-absorbed objects (and he did so much earlier than Kant's discussion on the “thing in itself”). Berkeley's philosophy of perception can be summarized in one sentence: To be is to perceive or be perceived *(Esse rerum est percipi*). Namely, to be is to be noticed. Once perception and being are equated, the world ceases to be made of things as autonomous beings.

Why should we call primary that which cannot be experienced? Once one commits to the distinction between primary and secondary qualities, conclusions are concealed in the premises. When perception is degraded in favor of measurement, experimentalists cease to be empiricists. Such strategy indeed creates an objective frame of knowledge. To say that space and time are the preconditions of experience is backwards. Experience and consciousness do not admit any mediator; they are given in immediacy. For Berkeley, the world presents to us in our perceptions, rather than being represented in them. We have been told repeatedly that our senses betray us. And that the tree would fall if nobody is looking at it. Leaving his extremely idealist position aside, “being as perceiving” has a major advantage: it can dispense with *simple location*. For Berkeley, perception is not in the subject who perceives, nor in the object perceived. It is neither in both at the same time, nor even between both. Perception is, on the contrary, what sustains them both. It is their foundation. From this worldview, the world is not made of “things,” but of perceptions, which are pointers to other perceptions. Things, being perceptions, are here and there at the same time. They are from where they look and in what they look.

As Borges remarked with unrivaled genius, there is that strange habit in which some qualities are considered substantives and other adjectives (Borges, 1925). And yet, nature is not static like a noun or secondary like an adjective, but durational like a gerund and circumstantial like an adverb. The object-subject distinction is disorienting. It already presupposes a metaphysics of differentiated subjects with privative predicates. “We find the world's contents grouped into things and their qualities” (Bradley, 1893, p. 19). Both, matter and mind, body and soul, are substantives “too big” for Borges.

We have seen how Whitehead's philosophy is tilted toward the radical empiricism of Berkeley or James, in which reality is identified with experience. He attributes experience to all things in the world. Berkeley had pointed in that direction, but no one like Whitehead had brought so far the identification of experience with reality. The implications of *pan-experientialism*, and its often-missed precise relation with pan-psychism (and the critiques therein) are beyond the scope of the present manuscript. If one claims that all is perception, one is soon haunted by the doubt about who sustains the tree that nobody sees. We do not need to suppose a God that sees it and sustains it, nor to admit that the tree disappears. Those who perceive it hold the tree. The earth feels the roots, and the wind the leaves, and the nest the branch.

Whitehead coined the term “eternal object” (which we cannot explain here due to its technicality) to distance himself from the concept of essence. His philosophy is a critique of modern philosophy, from Descartes to Kant, which has interpreted nature and the human being through the category of substance, justifying in this way the reproach to build a solipsist perspective, rather than understanding all real essences as subjects, which is the position that Whitehead adopts and that he calls the “reformed subjectivist principle.” The successful defect of the physical-mathematical scheme of the seventeenth century was to decide that reality is made of substances of independent existence. This was the starting point of scientific materialism, which gave way to mechanicism. The notion of simple location is a Newtonian mirage. The classical substance is self-contained, and it cannot be “in” another substance. The real, the concrete, is a continuous process of self-identity. Entities penetrate one another. They are in themselves and in other identities.

In sum, what happens when we bite an apple and experience its flavor? Berkeley would suggest that the flavor of the apple is not to be found in the apple itself, nor in the person that tastes it, but in the gathering of both (Berkeley, 1710, I.1). Here we have argued that this is not only applicable to flavor, but that it can be extended to a wide range of perceptions and thoughts. An apple is also the confluence of a seed, a tree, the rain, and the harvest. What we call “things” are actually processes. Things are encounters. Identities are crossroads. A flavor is not different from that other encounter we call a person. The things we perceive and imagine are gatherings and they have a provisional character. Such essential conditionality is what Buddhists call emptiness. Accordingly, one cannot say that the truth of the fugacity of things is an eternal truth, otherwise it would transform it into a product of the same kind of error as that which it denounces. The truth of the provisionality of identities is itself provisional and gets involved with a certain character of irony (passing truth has a soothing effect on imagination). The core of the problem of identity is that A = A is either a truism or false.

In fact, one of the most original ideas of the ancient Mahayana Buddhism (Nāgārjuna, 2011, 2.19, 6.4–5, 10.16, 20.19–20) was its critique of the notion of identity: there are not two identical things in nature; nothing is identical to another thing. According to this view, identity is impossible (A=A is a fallacy). If one cannot find in the world two equal beans, two exactly same cogs, even less two identical hopes or living beings. Not only there are no two equal grasshoppers, but, since they live in time, each grasshopper could never be identical to itself. The person that started to read this paragraph is not the same as the one who finishes it.

Berkeley was discarded too precipitately. And Whitehead's philosophy is still ungrasped. Whiteheadean or not, our exploration of *inter*-identities beyond reductionistic and mechanistic stances (even when covert in organicism) suggests to rather conceive them as *intra*-identities. We have a fascinating challenge: to be able to think of relations not between but *within*.

## Author Contributions

AG-M and JA conceived the project and wrote a first draft of the manuscript. AG-M wrote the full and final version of the manuscript. All authors contributed to the article and approved the submitted version.

## Conflict of Interest

The authors declare that the research was conducted in the absence of any commercial or financial relationships that could be construed as a potential conflict of interest.

## Footnotes

^1^Let us note that, first, internal relations do not denote a contrast between inside and outside (internal vs. external), but between intrinsic vs. extrinsic. Second, Moore discussed *internal relations* before Whitehead, but not in the same sense (Moore, 1919). Third, one must be cautious with some definitions of the notion of *internal relation*, especially when provided by analytic philosophers, influenced by Russell (see for instance Stanford Encyclopedia for Philosophy about internal and external relations). He thought that internal relations determine the related beings and thus make freedom impossible. Russell favors external relations, but he did not think in terms of transformation of essence. Yet, self-determination of human essence through internal relations to other essences is arguably the highest form of freedom.

^2^“Al descender hasta los microprocesos nos ha fallado el supuesto substancial; pero como íbamos cabalgando sobre los mismos procesos, haciendo caso omiso de su relación con una substancia, resulta que no nos hemos dado cuenta del cambio de corcel. Hemos abandonado la substancia y nos hemos quedado con la sola función. La sorpresa surge cuando se quiere atribuir substancialidad a los que eran simples comportamientos, o mejor dicho, meros cambios de propiedades de unas remotas substancias que ya no estaban inmediatamente ‘sustentando' los procesos elementales” (Panikkar, 1961, p. 281).
